# OptCircuit: An optimization based method for computational design of genetic circuits

**DOI:** 10.1186/1752-0509-2-24

**Published:** 2008-03-03

**Authors:** Madhukar S Dasika, Costas D Maranas

**Affiliations:** 1 Department of Chemical Engineering, The Pennsylvania State University, University Park, PA 16802, USA

## Abstract

**Background:**

Recent years has witnessed an increasing number of studies on constructing simple synthetic genetic circuits that exhibit desired properties such as oscillatory behavior, inducer specific activation/repression, etc. It has been widely acknowledged that that task of building circuits to meet multiple inducer-specific requirements is a challenging one. This is because of the incomplete description of component interactions compounded by the fact that the number of ways in which one can chose and interconnect components, increases exponentially with the number of components.

**Results:**

In this paper we introduce OptCircuit, an optimization based framework that automatically identifies the circuit components from a list and connectivity that brings about the desired functionality. Multiple literature sources are used to compile a comprehensive compilation of kinetic descriptions of promoter-protein pairs. The dynamics that govern the interactions between the elements of the genetic circuit are currently modeled using deterministic ordinary differential equations but the framework is general enough to accommodate stochastic simulations. The desired circuit response is abstracted as the maximization/minimization of an appropriately constructed objective function. Computational results for a toggle switch example demonstrate the ability of the framework to generate the complete list of circuit designs of varying complexity that exhibit the desired response. Designs identified for a genetic decoder highlight the ability of OptCircuit to suggest circuit configurations that go beyond the ones compatible with digital logic-based design principles. Finally, the results obtained from the concentration band detector example demonstrate the ability of OptCircuit to design circuits whose responses are contingent on the level of external inducer as well as pinpoint parameters for modification to rectify an existing (non-functional) biological circuit and restore functionality.

**Conclusion:**

Our results demonstrate that OptCircuit framework can serve as a design platform to aid in the construction and finetuning of integrated biological circuits.

## Background

Recent years has witnessed an increasing number of studies on constructing simple synthetic genetic circuits that exhibit desired properties such as oscillatory behavior, inducer specific activation/repression, etc. The hope is that these simple circuits are the vanguards of more complex ones with far ranging implications to biotechnology and medicine bringing to fruition the promise of synthetic biology. It has been widely acknowledged that that task of building circuits to meet multiple inducer-specific requirements is a challenging one [1-5]. This is because of the incomplete description of component interactions compounded by the fact that the number of ways in which one can chose and interconnect components, increases exponentially with the number of components. To meet these emerging challenges, in this paper we introduce an optimization based framework that, given an underlying quantitative description, automatically identifies the circuit components from a list and connectivity that brings about the desired functionality.

To date, several small synthetic gene networks that accomplish a specific functionality have been constructed. For example, Gardner and co-workers have developed a genetic toggle switch- a synthetic gene regulatory network that exhibits bistability [6]. Similarly, Elowitz and Leibler have constructed a synthetic circuit termed as repressilator that was designed to produce an oscillatory response [7]. Subsequently, researchers have extended the repressilator circuit design to induce synchronous oscillations [8], design a synthetic gene-metabolic oscillator [9] and many others [10-14]. Several researchers have employed synthetic circuits to investigate the dynamics and inner workings of more complex natural genetic networks. For example, Hooshangi et al. have constructed synthetic transcriptional cascades to investigate the ultrasensitivity and noise propagation in genetic networks [15]. Mangan et al. have investigated the structure and dynamics of the widely occurring feed forward loop motif [16,17]. Similarly, Becskei and Serrano designed simple gene circuits to examine the effects of autoregulation in gene networks [18].

In addition to uncovering the design principles of natural genetic networks, synthetic genetic networks are now increasingly finding roles in applications ranging from biotechnology, medicine and bio-sensing. For example, Martin et al. have successfully expressed enzymes from plants, yeast and *Escherichia coli *to produce amorphadine, a precursor to an anti-malarial drug artemisinin [19] and Anderson et al. have engineered the interaction between bacteria and cancer cells to depend on heterologous environmental signals [20]. Similarly, Levskaya et al. have devised a synthetic circuit that switches between different states in response to red light [21]. These ever-expanding applications have spurred the interest for the development of efficient experimental, database and computational techniques to support these efforts [22].

In response to these developments the research community has been rapidly moving towards standardization by creating the Registry of Standard Biological parts [23]. This registry provides a comprehensive compilation of well defined elements of a genetic circuit such as promoters, ribosome binding sites, transcripts, inducer molecules, terminator sites and plasmids among others. The impetus is that these spare parts registries will help usher the development of more rational engineering approaches for designing such circuits. The potential of using modeling and computations to better understand the function of these circuits has already been recognized and mathematical models have been proposed to describe the interactions between genetic elements [24-27].

The recent availability of well-defined spare parts lists and their interactions brings at the forefront the need to develop procedures to design and optimize genetic circuits that exhibit a desired functionality. Previous efforts in this direction include electrical circuit inspired designs proposed by Basu and Weiss [28,29]. By constructing a library of cellular gates the authors have implemented simple logical functions such as OR, NOT and AND. Similarly, Mason et al. have investigated the behavior of an electronic model of a gene circuit to produce oscillatory behavior [30]. Other efforts include the combinatorial synthesis approach employed by Guet et al. [31]. In this work the authors varied the connectivity of genes and their corresponding promoters thus generating an ensemble of responses from the resulting genetic circuits. This approach, however, becomes intractable for circuits involving a large number of components [2]. Another important consideration associated with the design and fabrication of genetic circuits is the proper matching of kinetic rates of individual elements of the circuits. Several studies have reported that failure to generate the correct response is often due to improper assembly of the basic elements. For example, simulations conducted by Tuttle et al. have confirmed that repressillator circuits constructed by using wild-type promoters do not result in oscillations [32]. Similarly, studies conducted by Hoosangni et al. have revealed that the behavior of a transcriptional cascade depends on the promoter leakiness and expression levels at the previous stage [15]. Several researchers have stressed the need for optimizing the kinetic parameters to ensure functionality and both experimental [33] and computational approaches [2,34,35] have been proposed to this end.

**Figure 1:**
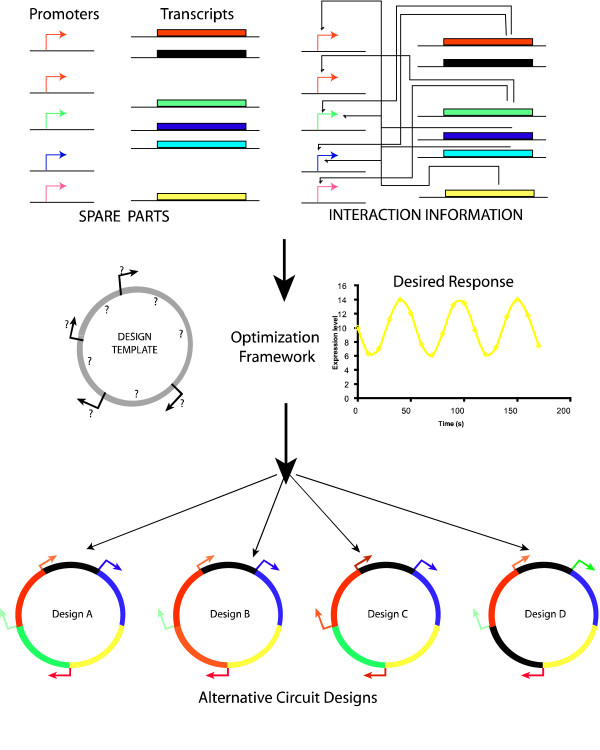
A pictorial illustration of the OptCircuit framework. The three key components of the framework are the basic genetic elements (promoters, transcripts, inducers); the underlying kinetic mechanisms that drive the circuit response and finally the desired behavior of the circuit under construction. These three components are integrated by OptCircuit using an optimization based formulation.

To address these questions, in this work we introduce OptCircuit (see Figure 1), an optimization-based framework that (i) automatically identifies the circuit components from a list and connectivity that brings about the desired functionality; (ii) Rectify or redesign an existing (non-functional) biological circuit and restore functionality by modifying an existing component (e.g., through changes in kinetic parameters) and/or identifying additional components to append to the circuit; Multiple literature sources are used to compile a set of kinetic descriptions of promoter-protein, protein-protein and protein-inducer pairs. The dynamics that govern the interactions between the elements of the genetic circuit are currently modeled using deterministic rate equations but the framework is general enough to accommodate stochastic simulations. The desired circuit response is abstracted as the maximization/minimization of an appropriately constructed objective function. Subsequently, an iterative procedure is implemented within our framework to identify an ensemble of circuits that exhibit the desired response. OptCircuit has been applied to a variety of applications ranging from the design of circuits that discriminate between inducer molecules; circuits that detect the combination of inducer molecules (i.e., 2 to 4 genetic decoder) and finally circuits whose responses are dependent on the concentration of the external inducer (concentration band detector).

## Results

In this section we highlight the capabilities of the OptCircuit framework to design circuits of varying stimulus and complexity. We first examine the design of simple circuit(s) against known architectures that discriminate between inducer molecules. Next, we dialup the complexity of the desired circuit response by seeking circuit configurations that can detect which combination of inducer molecules are present/absent (genetic decoder example). Finally, we test the ability of the framework to identify circuits whose responses are not only dependent on the presence/absence but also on the level of external inducers (concentration band detector example). In addition to identifying optimal configuration of the design variables, in the last example we also explore whether optimizing the kinetic parameters of specific elements within a given circuit can improve its performance.

### Inferring circuits with inducer-specific responses

Here we test OptCircuit by generating circuit designs whose responses are contingent on the presence/absence of different inducer molecules and compare the results with known designs [6]. Specifically, in the presence of anhydrotetracyclin (aTc) the desired circuit must express only protein *lacI *while in response to inducer IPTG the circuit must express only protein *tetR*.

The desired circuit response is imposed by maximizing the scaled difference between the expression of the desired minus the undesired florescent protein in response to the two different inducers in line with the description provided in the methods section.

$$Maximize\text{ Z}=\left(\left(\frac{P_{lacI}^{aTC}-P_{lacI}^{IPTG}}{P_{lacI}^{aTC}}\right)+\left(\frac{P_{tetR}^{IPTG}-P_{tetR}^{aTc}}{P_{tetR}^{IPTG}}\right)\right)/2$$

In Eq 1.1, $P_{lacI}^{aTC},P_{tetR}^{aTC}$ represent the levels of transcripts *lacI *and *tetR *in presence of inducer *aTc *and similarly, $P_{lacI}^{IPTG},P_{tetR}^{IPTG}$ represent the levels of *lacI *and *tetR *in presence of inducer *IPTG *respectively.

**Figure 2:**
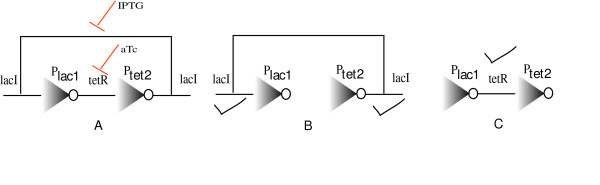
Shown in (A) is a relatively simple circuit identified by OptCircuit. Interestingly the circuit is reminiscent of the genetic toggle switch. Similar to the behavior of the toggle switch, in presence of aTc, the activity of *tetR *protein is suppressed. This enables the expression of *lacI *as shown in B. Finally, as shown in (C) in presence of IPTG, activity of *lacI *is suppressed which enables the expression *tetR *from the *P*_*lac *_promoter. The triangles with open circles at the vertices represent the promoter elements.

**Figure 3:**
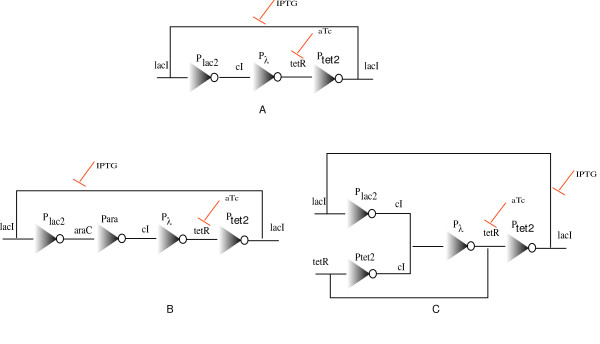
Alternative circuit configurations proposed by OptCircuit for the first example. OptCircuit is able to identify more complex architectures to realize a particular outcome. The triangles with open circles at the vertices represent the promoter elements.

Using OptCircuit we identify multiple circuits with up to two promoter transcript pairs. The circuit configuration for the best solution is shown in Figure 2A. Interestingly, the configuration is reminiscent of the architecture of the well-studied genetic toggle switch [6]. Briefly, in line with the construction of a genetic toggle switch, in presence of aTC, the activity of protein tetR is suppressed (see Figure 2B). This in turn leads to the expression of protein lacI from P_tet2_ promoter (since tetR suppresses expression from P_tet2 _promoter) as shown in the Figure 2B. On the other hand, in presence of inducer IPTG, the activity of protein lacI is suppressed which enables expression tetR from P_lac1 _promoter (see Figure 2C). In terms of computational requirements, a total of 50 iterations required a total of 200 minutes when run on a 16 node LINUX cluster running dual Intel 3.4 GHz Xeon processors. After performing an exhaustive search on circuits having two-promoter transcript pairs, the effect of dialing up the complexity of the designed circuits by allowing for as many as three to four promoter transcript pairs is examined. Our results shown in Figure 3, indicate that in addition to relatively simple circuit designs, akin to known ones, OptCircuit suggests non-intuitive designs with added complexity affording more opportunities for kinetic parameter tuning.

### Design of genetic decoder

**Figure 4:**
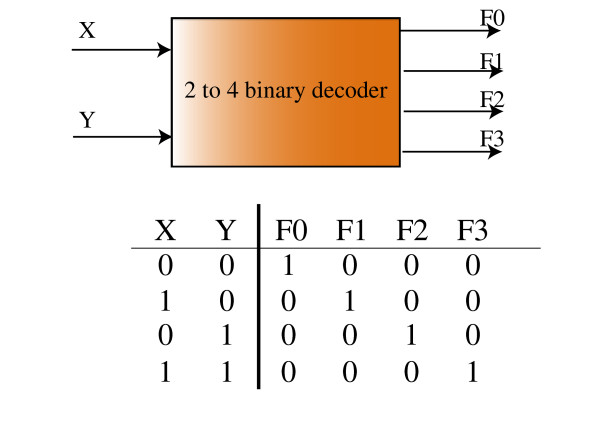
A pictorial illustration of the 2–4 bit decoder. In (A), a block diagram for a digital decoder is shown. In (B), the corresponding truth table associated with the decoder shown is illustrated. For the genetic decoder, X and Y represent *glucose L-arabinose *respectively. Further, F2, F0, F3 and F1 represent YFP, RFP, BFP and GFP responses respectively.

In this section, we use OptCircuit to design for more complex responses by constructing a genetic circuit equivalent of a 2–4 bit decoder. A digital decoder is a multiple-input, multiple-output logic circuit that converts coded inputs into coded outputs. Figure 4(a) illustrates the block diagram of a digital decoder and the corresponding truth table is shown in Figure 4(b). In the context of genetic circuits, we seek the design of a circuit architecture that produces four different responses dependent on the presence and/or absence of the sugars *glucose *(X in Figure 4) and *L-arabinose *(Y in Figure 4) respectively. Specifically, we would like the circuit to express (i) *YFP *(F2 in Figure 4) in response to the presence of *L-arabinose *and absence of *glucose*, (ii) *RFP *(F0 in Figure 4) in response to the absence of both *glucose *and *L-arabinose*, (iii) *BFP *(F3 in Figure 4) when both *L-arabinose *and *glucose *are present, (iv) *GFP *(F1 in Figure 4) when *L-arabinose *is absent but *glucose *is present. Note that since absence of glucose is known to induce the expression of cAMP, in this work we assume that absence of glucose implies presence of cAMP and equivalently, presence of glucose implies absence of cAMP.

**Figure 5:**
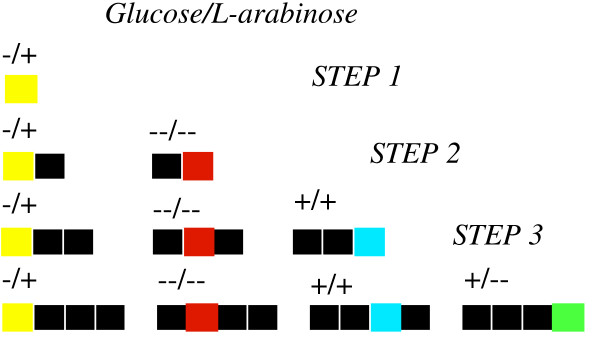
Step wise procedure to design genetic decoder. In the first step, circuit designs to produce YFP in absence of glucose and presence of L-arabinose are identified. Next, in step 2, additional elements are appended to the design from the previous step to produce YFP under (-/+) condition and RFP under (-/-) condition. Similarly at step 3 BFP response in introduced. Finally, additional elements are appended to the best designed identified by OptCircuit at step 3 to accomplish the response shown in step 4.

**Figure 6:**
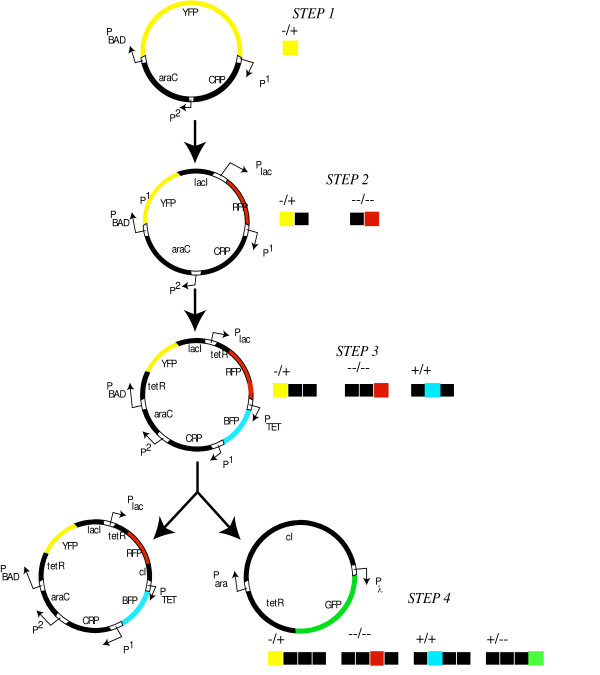
The circuit configurations identified by OptCircuit for the genetic decoder example. Note that the final step (step 4) is shown as a combination of two architectures.

Given *N *different promoter elements and *M *transcripts, the total number of design configurations with upto *K *promoter-transcript pairs is given by *(NM)*^*K*^. This implies that the search space characterizing all circuit configurations is enormous even for relatively modest values for *N *and *M *thus preventing its exhaustive navigation. To alleviate this problem, we implemented the OptCircuit framework in a sequential fashion where successive elements are appended to the genetic circuit to meet, one at a time, the four desired responses (see Figure 5). As shown in the Figure, at each step, the objective function values of the ten best circuit architectures are recorded and the circuit producing the best objective value is retained for the next step. The first step shown in Figure 5, involves the expression of *YFP *under the (-/+) (i.e. absence of *glucose *and presence of *L-arabinose*) condition. To this end, we borrowed the circuit configuration from the well studied, feed-forward loop architecture [16,17]. *CRP *and *AraC *are expressed from the constitutive promoters, $P_{cons}^{1}$ and $P_{cons}^{2}$ respectively and *YFP *is placed under the control of the *P*_*BAD *_promoter. Using this as the seed, the OptCircuit framework is employed to sequentially identify additional components by following the step-wise procedure shown in Figure 6. After the second step (i.e., (-/-) response), our framework identifies the expression of *lacI *from the *P*_*BAD *_promoter and the expression of *RFP *from the *P*_*lac *_promoter. In the third step (i.e., (+,+) response) the best objective value was realized following the expression of protein *tetR *from *P*_*BAD *_and *P*_*lac *_promoters and expression of *BFP *from the *P*_*tet *_promoter which is repressible by protein *tetR *(Figure 6 step 3). Finally after the last step ((+,-) response), by allowing for expression of *GFP*, the additional elements appended to design the decoder include, the expression of protein *cI *from the *P*_*ara *_and *P*_*lac *_promoters and the expression of proteins *tetR *and *GFP *from the *P*_*λ *_promoter.

**Figure 7:**
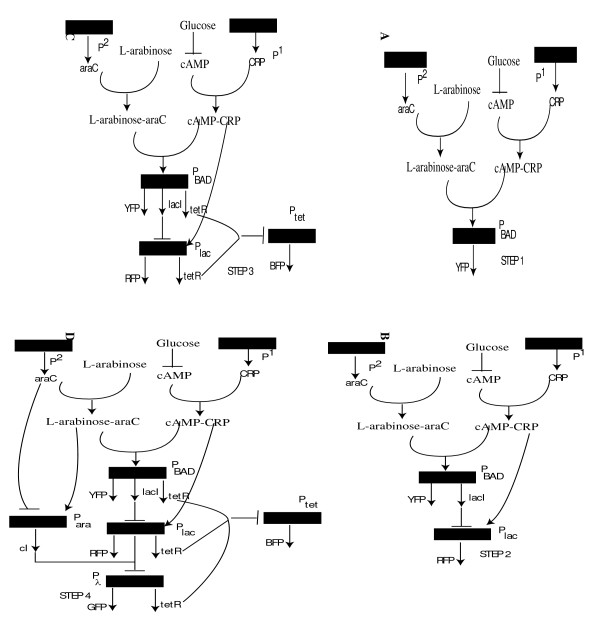
A network illustration describing the workings of the decoder at each step of the stepwise procedure. Activating interactions are represented by pointed arrows and inhibiting interactions are represented by arrows with flat heads. In (a), CRP-cAMP complex together with L-arabinose-araC complex activate expression of YFP from *P*_*BAD *_promoter under (-/+) condition. In (b),(c), (d), the networks at step 2, step 3 and step 4 are illustrated.

Figure 7(a–d) provides a network illustration describing the workings at each step of the genetic decoder. As shown in the Figure, in the final step, under (-/+) conditions, expression of YFP along with *lacI *and *tetR *is induced from *P*_*BAD *_promoter. Further, *araC-L-arabinose *complex activate expression of *cI *from *P*_*ara *_promoter which serves to shut OFF expression of GFP. Under -/- conditions, RFP is expression is induced as before; However, observe that *P*_*lac *_promoter induces expression of *cI *to shut down expression of GFP (see Figure 7d). Under (+/+) condition, BFP expression from *P*_*tet *_promoter is induced as in the previous steps and production of *cI *from *P*_*ara *_promoter prevents expression of GFP from *P*_*λ *_promoter. Finally, under (+/-) condition, *P*_*BAD*_, *P*_*ara *_and *P*_*lac *_promoters are shut OFF which in turn enables the expression of GFP from *P*_*λ *_promoter.

**Figure 8:**
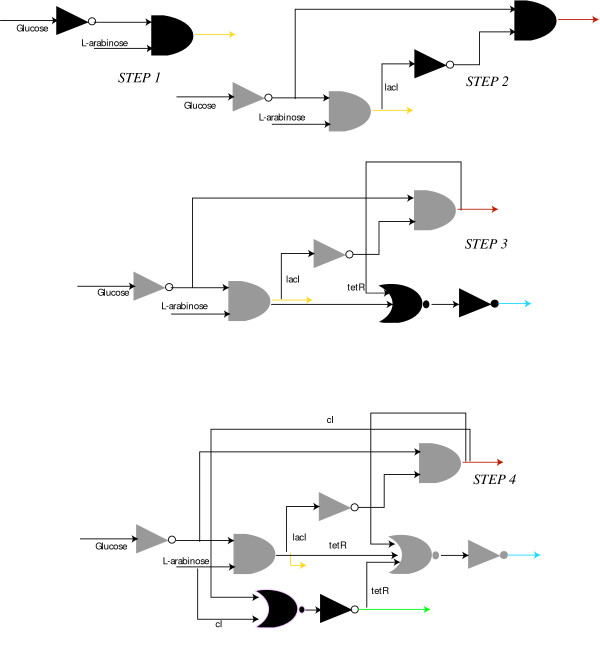
Binary logic representation of the circuits obtained in Figure 5. The additional elements appended at each step are indicated using black colored logic gates. Observe that at each step, the OptCircuit framework appends an OR gate followed by a NOT gate to generate the required response. The OR gate serves to integrate the indirect effect of the expression under corresponding inducer conditions. A legend describing the representation of the logic gates is presented in supplementary material (see Additional file 1, Figure S2).

The results shown in the previous paragraph also sheds light onto the design principles for the construction of a genetic decoder. Figure 8, illustrates the binary logic schematics for the genetic circuits characterizing each step shown in Figure 6 and Figure 7. Observe that at each step, the OptCircuit framework allows for the addition of components that are activated only when the corresponding inducer conditions are met. In addition, by expressing appropriate repressor molecules, the OptCircuit framework ensures the repression of all the other promoters expressing fluorescent proteins. For example, after Step 1 the AND gate expressing *YFP *is active only when *glucose *is absent of *L-arabinose *is present. Subsequently, a NOT gate logic is introduced after the second step to turn OFF the *P*_*lac *_promoter when *YFP *is expressed. After the third step, an OR gate with two inputs followed by an NOT gate is introduced. The OR gate combines the indirect repressive effect of *tetR *that turns OFF the production of *BFP *if either *YFP *or *RFP *are expressed. Finally, after the last step the OR gate with three inputs of *tetR *and and an OR gate with two inputs of *cI *followed by a NOT gate are introduced. This ensures that if either one of *BFP*, *RFP *or YFP are expressed then *GFP *is turned OFF and conversely *GFP *is expressed only if none of the other three reporters are expressed.

**Figure 9:**
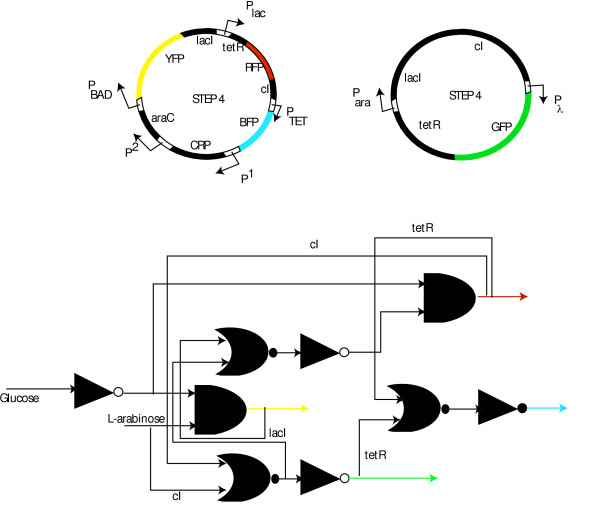
Alternative circuit design predicted by the OptCircuit framework. The corresponding binary logic diagram is also provided for comparison. The colored arrows indicate the expression of corresponding colored fluorescent proteins. The logic gates are represented using established conventions. The gates include, AND, the triangles represent NOT gates or inverters and the crescent shaped gates are the OR gates. A legend describing the representation of the logic gates is presented in supplementary material (see Additional file Figure S2).

**Figure 10:**
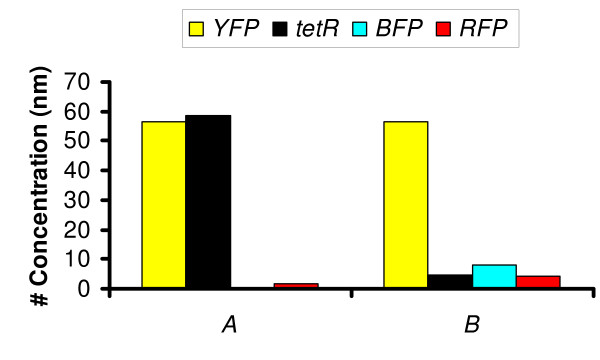
The steady-state levels of proteins for the circuits shown in Figure 6(step 4) and Figure 9. Circuit (A), represents the circuit shown in final step of Figure 6. (B) represents the circuit in Figure 9.

The identified circuit design (Figure 6) happens to be consistent with a purely binary logic viewpoint of regulation. This is not the case with all identified designs. For example, one such circuit configuration involves the expression of protein *lacI *from *P*_*ara *_promoter instead of the expression of protein *tetR *from the *P*_*BAD *_promoter (see Figure 9) leading to a behavior that is inconsistent with Boolean-only regulation. To illustrate this, consider the truth table of the design shown in Figure 9. Under -/+ condition *YFP *is expressed and *RFP *and *GFP *are shut-off. However, unlike the circuit described in previous paragraph, expression of *YFP *is not accompanied by expression of *tetR *and hence the *P*_*tet *_promoter is free to express the fluorescent protein *BFP*. Nevertheless, OptCircuit identified this circuit configuration as an optimal architecture for a genetic decoder because the employed kinetic description accounts for *not only the presence but also the level *of each participating molecule needed to activate transcription. Figure 10, provides a comparison of the steady-state levels of proteins *tetR *and *BFP *for the circuits described in Figure 6 (step 4) and Figure 9. In circuit (A), the level of *tetR *is relatively high (~60 nm) which in turn strongly represses the expression from the *P*_*tet *_promoter. This is expected since, in circuit A, expression of *YFP *is accompanied by expression of *tetR*. In contrast, in circuit B, even though the level of protein *tetR *is relatively low (~10 nm), examination of the level of protein *BFP *suggests that even low levels of protein *tetR *are able to effectively repress the expression of *BFP *from the *P*_*tet *_promoter. The low level of *tetR *is a manifestation of the leaky repression exerted on the *P*_*lac *_promoter by the *lacI *protein. This observation is further substantiated by expression of protein *RFP*, albeit at low levels. These results indicate that by taking into account the underlying kinetic description of the interactions, the OptCircuit framework is able to expand upon possible circuit designs by drawing from architectures that may not be valid based on digital logic viewpoint though adequately meet the imposed requirement due to the careful matching of kinetic parameters as often observed in nature.

### Design of Concentration Band Detector

**Figure 11:**
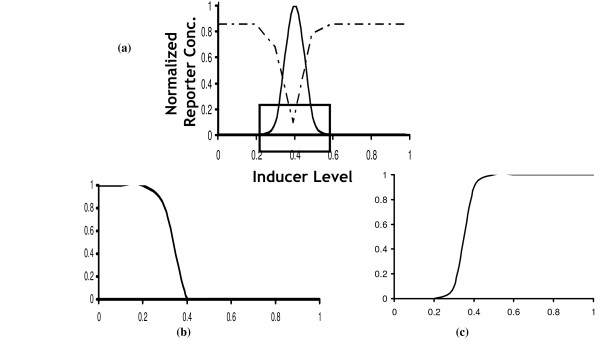
The desired response from the concentration band detector example. The solid line represents the desired response of the inducer molecule (*GFP*). The dotted line represents the desired response of the protein *tetR*. In (b), the desired response of protein *tetR *in the LTD circuit is shown, and in (c), the corresponding response form the HTD circuit is illustrated.

With this example, we explore whether OptCircuit can pinpoint design configurations whose responses are dependent not only on the presence/absence of external inducers but also on their concentrations. We use the concentration band detector example [36] to demonstrate the OptCircuit application. Briefly, this circuit expresses high levels of a reporter protein only when the concentration of the external inducer (i.e. *L-arabinose*) is within a specific range [36] (i.e., neither too high or too low) as shown in Figure 11.

In line with the design proposed by Basu and coworkers, OptCircuit first places the reporter protein under the control of a repressible promoter (i.e., P_*tet *_promoter) which is repressed by protein *tetR *(dotted line in Figure 11(A)). Subsequently, we use OptCircuit to design two circuits, a low threshold detector (LTD) and a high threshold detector (HTD). The LTD circuit expresses high levels of *tetR *at low levels of *L-arabinose *and low levels of *tetR *at high levels of *L-arabinose *(see Figure 11(B)). In contrast, the HTD circuit is designed to express low levels of *tetR *at low levels of inducer and high levels of *tetR *at high levels of *L-arabinose *(see Figure 11(C)). Finally, the LTD and HTD circuits are fused together to obtain an inverted bell shaped response for protein *tetR*.

**Figure 12:**
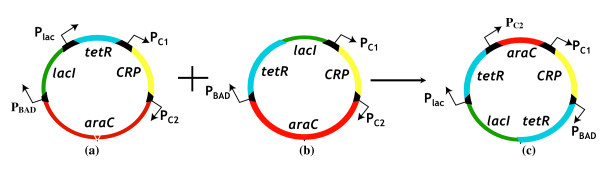
The best circuit configurations proposed by OptCircuit. In (A), the LTD circuit is shown; In (B), HTD circuit is illustrated and finally the combination of both the circuit designs is shown in (C).

**Figure 13:**
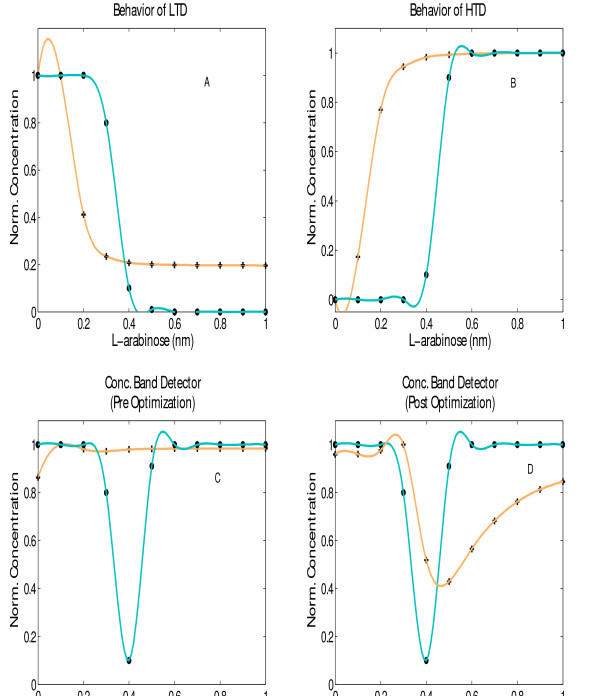
In (A), the response of the LTD circuit is shown. In (B), the response obtained from HTD is illustrated. In (C), the response obtained prior to optimization of kinetic parameters is shown. Note that this response deviates significantly from the desired response. In (D), the response obtained after optimizing kinetic parameters is shown. Specifically the efficiency of constitutive promoter expressing *CRP *is decreased 13 fold. In all the figures cubic splines were used to interpolate the observed steady-state expression levels.

The best circuit configurations proposed by OptCircuit are shown in Figure 12. The only difference between the LTD and HTD is that while *tetR *is expressed from the P_lac _promoter in the LTD, it is expressed from P_BAD _promoter in the HTD. Examination of the circuit behavior reveals that at low levels of *L-arabinose*, the P_BAD _promoter is not sufficiently activated ensuring low levels of protein *lacI*. This in turn implies that the P_lac _promoter is free to express *tetR *from the LTD circuit (see Figure 12 and Figure 13(A)). As the amount of *L-arabinose *accumulates in the system, the transcriptional expression from the P_BAD _promoter is enhanced leading to expression of *lacI *from LTD and *tetR *from HTD (see Figure 12 and 13(B)). Finally, expression of *lacI *from HTD turns off expression of *tetR *from HTD. The final OptCircuit design enables the expression of protein *tetR *from P_lac _and P_BAD _promoters, *lacI *from PBAD promoter and reporter protein *GFP *from P_tet _promoter. The level of protein *tetR *as a function of level of *L-arabinose *is shown in Figure 13(C). As shown in Figure 13(C), we find that the circuit response deviates significantly from the desired response implying that by simply reshuffling existing components the desired response is not attainable.

To address this remaining challenge we next explore whether modifying any existing component in the circuit will shift the circuit response closer to the desired response. Specifically, the circuit described in the previous paragraph is "fixed" and subsequently starting from the current parameter values as an initial guess we optimize the kinetic parameter values using Eq 1.13. Results indicate that a considerable improvement in circuit response is obtained when the transcriptional efficiency of the constitutive promoter expressing protein *CRP *is decreased 13 fold. This resulted in a 18.69% decrease in the objective value (8.1071 → 6.5976). The effect of this parameter modifications are quantified in Figure 13(D) demonstrating that OptCircuit can be used to pinpoint kinetic parameter modifications improving its functionality.

## Discussion

In this work, we introduced an optimization-based approach termed OptCircuit that (i) automatically identifies the circuit components from a list and connectivity that brings about the desired functionality; (ii) Rectify or redesign an existing (non-functional) biological circuit and restore functionality by modifying an existing component and/or identifying additional components to append to the circuit. The dynamics that govern the interactions between the elements of the genetic circuit were modeled using deterministic rate equations and the desired circuit response is abstracted as the maximization/minimization of an appropriately constructed objective function. Subsequently, an iterative procedure was implemented within our framework to identify an ensemble of circuits that exhibit the desired response. The capabilities of the developed tool were investigated by synthesizing circuits that exhibit a wide array of responses. The genetic toggle switch example demonstrated the ability of the framework to suggest simple or more complex circuit configurations capable of discriminating between inducer molecules. The 2 to 4 genetic decoder example led to complex circuit designs consisting of as many as 13 promoter-transcript pairs that may or may not be identifiable through a digital logic based design procedure. Finally, the concentration band detector example illustrated how OptCircuit can be used to design not the architecture of the synthetic circuit but also suggest modifications on its kinetic parameters for optimized performance. OptCircuit can also be employed in tandem with existing computational methods for fine-tuning circuit performance by providing initial configurations. For example, Feng and co-workers developed a global sensitivity analysis based approach to identify the optimal parameter configuration [2]. In their approach, they start from a representative circuit configuration and then proceed to identify the optimal parameter set by estimating the sensitivity of the parameter variation on the circuit response.

It is important to emphasize that all the kinetic parameter modifications suggested by OptCircuit can be realized using a host of experimental strategies. For example in the construction of repressilator circuit, the authors control the rate of protein degradation by *ssrA *tagging whereby an amino acid sequence is introduced into the proteins which makes them a target for all proteases [37]. Similarly Yokobayashi and co-workers have used directed evolution to restore the performance of an unoptimized circuit [33]. Specifically, by focusing on the *cI *gene and its corresponding ribosome binding site, the authors report mutations that potentially reduce the translational efficiency or reduce the ribosome binding affinity. Other promising strategies include the approaches developed by Lutz and Bujard [38] to control the promoter activity and repression for the P_tet _and P_lac _promoters.

While our results demonstrate the ability of the framework to design circuits that accomplish a wide variety of responses, it is important to emphasize here that the framework does not take into account potential interactions with other biological components and processes present in the cellular environment. Further, the deterministic nature of OptCircuit may render the circuit deign sensitive to stochastic fluctuations and hence may fail to function properly in wake of noise. This observation assumes further significance in light of multiple modeling studies that have demonstrated the strong influence of noise and stochastic events on circuit performance [15,39-43]. For example, examination of the decoder circuit response to varying level of input signal reveals that the while the circuit is optimal with respect to connectivity; it is sensitive to changes in the input signal levels (see Additional file 1, Figure S1). This implies that the circuit behavior can be realized in only a very narrow range of concentrations [44] motivating the need to design circuits that are inherently robust to noise and leakiness of specific components. Key features that confer robustness are redundancy, modularity and the ability to decouple perturbations [45]. While most of the current literature regarding biological robustness has focused on elucidating the architectural and mechanistic features of a network, much less effort has been devoted to developing quantitative and qualitative criteria for quantifying robustness. Efforts in this direction include the works of Cherry and Adler [46] and Tomshine and Kaznessis [41] who have proposed that large separation between steady states is likely to render the biological switch immune to stochastic fluctuations. Subsequently, H. El-Farra et al. [47] employed these performance measures to develop optimization problems to identify parameters that confer robustness. The above observations highlight one potential way of improving the robustness of the circuits using OptCircuit. Specifically, performance measures such as separation between steady-states could be imposed as appropriate formulated objective functions to systematically synthesize circuits that are likely to be robust to stochastic fluctuations. Similarly, enforcing alternative ways of realizing an outcome can incorporate other qualitative metrics such as redundancy. We are currently exploring ways of protecting against component failure and incorporating robustness into the circuits we developed in this manuscript.

Another important limitation of the current approach lies in the computational requirements to accomplish a specific circuit design. We find that while OptCircuit was able to readily design circuits with relatively simple architecture (genetic toggle switch), the design procedure for more complex responses (decoder and band detector) entails expensive computational resources. However, our results indicate that the computational burden may be alleviated to a certain extent by exploiting the inherent decomposable structure built into the circuit responses. Specifically, both the decoder and band detector circuits were constructed by assembling smaller less complex building blocks (e.g. LTD and HTD in concentration bad detector). These observations suggest that by carefully identifying key decomposable structures within the desired response, it is possible to overcome this important limitation of the framework.

## Conclusion

In recent years, researchers have deposited several standard and interchangeable biological parts in the registry of standard biological parts (e.g. composite parts such as Isoamyl alcohol generating device (BBa_J45400), Elowitz repressilator (BBa_I5610)).

Currently efforts are underway to specify the functionality of these parts in terms of parameter estimates and behavior. Our results and those proposed by other researchers conclusively demonstrate that proper parameter compatibility is essential to ensure funtionality. As the characterization of these parts is moving at a fast pace, the OptCircuit framework could serve as a design platform to aid in the construction and finetuning of integrated biological circuits.

## Methods

### Modeling framework

The basic elements constituting a genetic circuit include promoter elements, protein/transcript molecules and inducers. Briefly, promoters are regions of DNA where RNA polymerases bind to initiate transcription. Transcripts referred to ORFs which upon transcription and translation produce proteins which in synthetic circuits act as transcriptional regulators repressing or activating a promoter's strength. Finally, inducers are small molecules (e.g., aTC, IPTG) which by directly interacting with transcription factors can block, enable or simply modulate a transcriptional regulation event. The quantitative description of the mechanistic detail underlying the interactions embedded in the genetic circuitry requires the definition of the following sets and variables.

$$\begin{array}{ll} Sets: & Variables:\\ \begin{array}{l} I=\{i\}=\text{set of promoters}\\ J=\{j\}=\text{set of transcripts}\\ K=\{k\}=\text{set of inducers}\\ T=\{\text{t}\}=\text{time} \end{array} & \begin{array}{l} P_{j}(t)=\text{protein level of transcript }j\text{ at time }t\\ Y_{ij}=\{\begin{array}{l} 1\text{ if transcript }j\text{ is expressed from promoter }i\\ 0\text{ otherwise} \end{array} \end{array} \end{array}$$

The set *I *represents all the promoter elements investigated in this study. *J *represents the set of transcripts and finally *K *is the set of all inducer molecules. Model variables encode the structure of the synthetic circuit and quantify the protein levels. Specifically, the binary variable *Y*_*ij *_determines which transcript *j *is expressed from a promoter *i *and *P*_*j *_(*t*) quantifies the level of protein *j *at a given time *t*.

### Kinetic description of interactions

Genetic circuits are characterized by a number of interactions including protein-promoter and protein-inducer and protein-protein interactions. For example, protein *lacI*, in its tetramer form functions as a repressor for P_lac _promoter while inducer molecule aTc suppresses the activity of protein *tetR*. In genetic circuits, unlike digital or binary logic based circuits, the presence/absence of a particular set of interactions alone is insufficient to accurately predict correctly all possible responses. In fact, several studies have reported that in addition to interactions, the kinetic rates of individual elements have to accurately match in order to ensure function. To this end, the kinetic description of each element of a genetic circuit is embedded into the OptCircuit framework.

Specifically, for every transcript *j*, the set of ordinary differential equations (ODE's) that govern the time evolution of the protein is given by Eq 1.2.

$$\frac{dP_{j}}{dt}=\sum_{i}Y_{ij}[\text{Rate of Production of j from i}]-K_{decay}^{j}P_{j}(t)\forall j$$

The first term in Eq 1.2, accounts for the cumulative rate of production of a particular protein *j *from the promoter elements and the second term represents the first order decay of the protein. Also observe that the production of a protein *j *from a promoter *i *is turned ON if and only if the corresponding binary variable *Y*_*ij *_is equal to one.

OptCircuit accounts for the activating and repressing effects on every promoter *i *within the framework by using the modeling formulation proposed by Hasty et al. [25]. Briefly, all biochemical reactions characterizing the interactions affecting a particular promoter are listed and divided into fast and slow steps. The fast reaction set typically includes protein dimerization and protein promoter binding while transcription and degradation steps compose the slow reaction set. The dynamics governing the promoter kinetics are derived using mass action kinetics with fast reactions that have rate constants in the order of seconds, assumed to be in equilibrium [25]. The modeling environment in OptCircuit is versatile enough to incorporate finer levels of mechanistic detail whenever available (e.g., modeling of mRNA [7]). The complete list of kinetic formulations for the promoter elements and the corresponding kinetic parameters employed in this study are provided in the supplementary material (see Additional file 1).

### Objective Function Modeling

The reliance on an optimization framework for designing synthetic circuits with a desired response implies that the objective function must be carefully chosen so as its maximization or minimization is a good surrogate of the desired response(s). The type of desired responses is partitioned into inducer-free and inducer-dependent ones. Inducer-free responses translate into the design of circuits whose response is consistent with a targeted time-course. This response may be oscillatory, constant or ramping up/down. For all these case the objective function, *Z *minimizes the sum of the squared departures from the targeted responses at all time points:

$$Minimize\text{ Z}=\sum_{t}{\left(P_{j^{*}}(t)-P_{j^{*}}^{\exp}(t)\right)}^{2}$$

In Equation 1.3, $P_{j^{*}}^{\exp}(t)$ denotes the experimentally observed profile. Inducer-dependent responses require a clear distinction between states corresponding to presence/absence of multiple inducers. Specifically, to accomplish this, an objective function is constructed as follows that maximizes the scaled separation between the inducer-present/absent responses:

$$Maximize\text{ Z}=\sum_{\text{j}\in \text{R}}\sum_{k\in K}\frac{{P_{j}(T)|}_{k}-\sum_{j^{\prime }\neq j\in R}{P_{j^{\prime }}(T)|}_{k}}{P_{j}(T){|}_{k}}$$

In Equation 1.4, *K *represents the set of inducer molecules present in the system, *R *represents the set of reporter proteins (e.g. *GFP*, *YFP *etc) and *P*_*j*_(*T*)|_*k *_represents the steady state levels of transcript *j *in presence of inducer *k*. Alternatively, if the circuit response must be inducer concentration dependent then the objective function can be formulated again as a the minimization of a least squares sum by considering multiple inducer concentrations.

$$Minimize\text{ Z}=\sum_{r}{\left({P_{j}|}_{k,r}-{P_{j}^{\exp}|}_{k,r}\right)}^{2}$$

In Eq 1.5, *r *represents the discretizations levels for the inducer concentration. *P*_*j*_|_*k*, *r *_and ${P_{j}^{\exp}|}_{k,r}$ represent the simulated and the desired steady-state levels of reporter transcript *j *at inducer discretization level *r*. These are only some examples of desired circuit responses. Using this optimization-based framework even more complicated responses can be modeled limited only by the imagination of the circuit designer. In the examples investigated in this study Eq 1.4 represents the objective function imposed for the genetic decoder example and Eq 1.5, for the concentration band detector example.

### Optimization model

Using the notation listed above, the problem of designing a genetic circuit that exhibits a desired response is formulated as the following mixed integer dynamic optimization problem (MIDO) [48-52].

Min/Max Z = *f *(*P*_*j *_(*t*))

*s*.*t*.

$$\frac{dP_{j}}{dt}=\sum_{i}Y_{ij}[\text{Rate of Production of j from i}]-K_{decay}^{j}P_{j}(t)\forall j$$

$$\begin{array}{cc} \sum_{j}Y_{ij}\leq P^{\max} & \forall i \end{array}$$

$$\begin{array}{cc} \sum_{i}Y_{ij}\leq T^{\max} & \forall j \end{array}$$

$$\begin{array}{c} \sum_{i}\sum_{j}Y_{ij}\leq M_{Max} \end{array}$$

The objective function in Equation 1.6 models the circuit response imposed by the circuit designer. Equation 1.7, describes the time evolution of protein levels as a set of ordinary differential equations as described in the previous section. Equation 1.8 imposes an upper limit on the number of transcripts a particular promoter *i *can express. Similarly, Equation 1.9 imposes a limit on the number of times a particular transcript *j *can be expressed from different promoters. Finally, Equation 1.10 imposes a limit on the total number of promoter-transcript pairs in the designed genetic circuit.

The boolean constraints (Equations 1.8–1.10) offer the flexibility to incorporate the design of an existing biological circuit and probe its behavior. This can be accomplished by incorporating constraints of the form

*Y*_*ij *_= 1 ∀ ({i, j} ∈ *EX*)

where, the set *EX *contains the connectivity information of the circuit. This feature confers upon us the ability to readily extend the framework to rectify or redesign an existing (non functional) biological circuit by identifying additional components to append to the circuit to ensure its functionality.

The solution procedure for the MIDO class of optimization problems is difficult [52] due to the simultaneous presence of binary variables *Y*_*ij *_and constraints in form of ODE's. Reliable solution methodologies that guarantee a global optimal solution for this class of problems are still in infancy [53]. Therefore, in this paper we rely on a decomposition procedure to bracket an optimal solution. The basic idea of proposed approach is to generate a converging sequence of upper and lower bounds to the original problem. The solution procedure is listed in a step-wise manner below.

**Step 1: **Initialize iteration counter, *iter *= 1; SET upper bound UB = ∞; SET lower bound LB = -∞; Generate an initial guess for a feasible circuit design $Y_{ij}=Y_{ij}^{iter}$. In this implementation of the framework, the initial guess is generated by simply solving the following optimization problem with a objective function set to zero.

Min Z = 0

*s*.*t*.

$$\begin{array}{cc} \sum_{j}Y_{ij}\leq P^{\max} & \forall i \end{array}$$

$$\begin{array}{cc} \sum_{i}Y_{ij}\leq T^{\max} & \forall j \end{array}$$

$$\begin{array}{c} \sum_{i}\sum_{j}Y_{ij}\leq M_{Max} \end{array}$$

After convergence to local optima, this initial guess is excluded by using integer cuts and the above problem is used to generate the next starting point.

**Step 2: **Integrate the system of ordinary differential equations (1.2) for fixed values of the design variables $Y_{ij}=Y_{ij}^{iter}$ to obtain the objective function value *Z*; SET UB = min(UB,Z). Store the solution corresponding to the best upper bound.

**Step 3: **In step2, we compute an upper bound for the objective function value. In step3 we compute a lower bound. The main ideas involved in the computation of the lower bound are presented in the development of the outer approximation procedure for solving non-linear and mixed integer non-linear optimization algorithms [54]. Briefly, the Master problem described in step 3 of the optimization procedure constructs a relaxation of the original feasible space by constructing supporting hyper planes at the point of interest. It has been shown previously that the solution to the Master problem yields a lower bound to the objective function value [54]. In our case the supporting hyper planes are constructed at the integer solution(s) obtained from step 2 and the partial derivates are computed by finite difference method. The master problem yields a lower bound on the objective function value and a new integer solution. A brief description of the main ideas governing the outer approximation procedure is provided in supplementary material (see Additional file 1).

Construct the master (lower bounding) problem as follows.

min *imize μ*

*s*.*t*.

$$\begin{array}{cc} \mu \geq Z^{iter}+\sum_{i}\sum_{j}\left(Y_{ij}-Y_{ij}^{k}\right){\left(\frac{\partial Z}{\partial Y_{ij}}\right)}_{Y_{ij}=Y_{ij}^{k}} & \forall k=1,2,...iter \end{array}$$

$$\begin{array}{cc} \sum_{j}Y_{ij}\leq P^{\max} & \forall i \end{array}$$

$$\begin{array}{cc} \sum_{i}Y_{ij}\leq T^{\max} & \forall j \end{array}$$

$$\begin{array}{c} \sum_{i}\sum_{j}Y_{ij}\leq M_{Max} \end{array}$$

The partial derivates are computed using finite difference methods.

Solve the master problem to obtain the objective function value μ* and integer solution, $Y_{ij}^{*}$.

SET LB = μ*

**Step 4: **If LB ≥ UB, then STOP (crossover). Otherwise, increase iteration counter *iter *→ *iter *+ 1. $Y_{ij}^{iter}=Y_{ij}^{*}$. Return to Step 2.

In addition to identifying the optimal configuration of design variables ($Y_{ij}^{*}$), OptCircuit can also be employed to optimize kinetic parameters of specific elements within the genetic circuit. For example, given a genetic circuit, the task of determining the optimal promoter strength of a particular promoter i* can be achieved by replacing Equation 1.12 with

$$\begin{array}{cc} \mu \geq Z^{iter}+\left(\alpha_{i}-\alpha_{i}^{k}\right){\left(\frac{\partial Z}{\partial \alpha_{i}}\right)}_{\alpha_{i}=\alpha_{j}^{k}} & \forall k=1,2,...iter \end{array}$$

Note that given the nonlinear nature of the problem under investigation the above procedure is carried out multiple times starting for several starting initial guesses and the local optimum solution identified at each iteration is stored along with a sorted list of the best circuit configurations.

## Authors' contributions

MSD and CDM designed and developed the computational algorithms. MSD performed the experiments. MSD and CDM wrote the manuscript. Both the authors have read and approved the final manuscript.

## Supplementary Material

Additional file 1 Supplementary material. This file provides the following information. i) List of promoters, transcripts and inducer molecules employed in this study. ii) A description of the activating and inhibiting interactions employed in this work. iii) The set of ordinary differential equations used for the genetic toggle switch example. In addition a brief description of the mechanistic detail embedded in these equations is provided. iv) List of nominal parameter values that were used for the genetic toggle switch example (1^st ^example). v) Equations describing the production terms for the promoters used for the genetic decoder and concentration band detector examples. vi) List of nominal parameter values that were used for the genetic decoder and concentration band detector examples (2^nd^/3^rd ^examples). vii) The sensitivity of the reporter proteins to varying levels of input signals in the genetic decoder example. viii) Legend describing the representation adopted for the logic gates. ix) A brief description of the main ideas behind the outer approximation procedure. Click here for file
