# Supplementary material for: OptCircuit: An optimization based method for computational design of genetic circuits
Source: BMC Syst Biol. 2008 Mar 3;2:24. doi: 10.1186/1752-0509-2-24 (PMC2324073; doi:10.1186/1752-0509-2-24)
Supplement: Additional file 1 — Supplementary material. This file provides the following information. i) List of promoters, transcripts and inducer molecules employed in this study. ii) A description of the activating and inhibiting interactions employed in this work. iii) The set of ordinary differential equations used for the genetic toggle switch example. In addition a brief description of the mechanistic detail embedded in these equations is provided. iv) List of nominal parameter values that were used for the genetic toggle switch example (1st example). v) Equations describing the production terms for the promoters used for the genetic decoder and concentration band detector examples. vi) List of nominal parameter values that were used for the genetic decoder and concentration band detector examples (2nd/3rd examples). vii) The sensitivity of the reporter proteins to varying levels of input signals in the genetic decoder example. viii) Legend describing the representation adopted for the logic gates. ix) A brief description of the main ideas behind the outer approximation procedure. [file 1752-0509-2-24-S1.doc]

**Supplementary Table 1**: This table provides a list of promoters, transcripts and inducer molecules employed in this study.

**Supplementary Table 2**: This table provides a description of the activating and inhibiting interactions employed in this work.

**Supplementary Information1**: The set of ordinary differential equations used for the genetic toggle switch example. In addition a brief description of the mechanistic detail embedded in these equations is provided

**Supplementary Table 3**: This table provides a list of nominal parameter values that were used for the genetic toggle switch example (1^st^ example)

**Supplementary Information2**: Equations describing the production terms for the promoters used for the genetic decoder and concentration band detector examples.

**Supplementary Table 4**: This table provides a list of nominal parameter values that were used for the genetic decoder and concentration band detector examples (2^nd^/3^rd^ examples)

**Supplementary Figure 1:** The sensitivity of the reporter proteins to varying levels of input signals in the genetic decoder example.

**Supplementary Figure 2:** Legend describing the representation adopted for the logic gates.

**Supplementary Information 3:** A brief description of the main ideas behind the outer approximation procedure.

| **PROMOTERS TRANSCRIPTS INDUCERS** |
| --- |
| P_lac1_ tetR aTc  P_lac2_ lacI IPTG  P_lac3_ cI cAMP/glucose  P_lac4_ araC L-arabinose  P_λ_ GFP  P_tet1_ YFP  P_tet2_ RFP  P_ara_ BFP  P_BAD_ CRP  P^1^  P^2^ |

**Table 1:** List of promoters transcripts and inducers used in this study.

| **PROMOTERS REPRESSOR ACTIVATOR** |
| --- |
| P_lac1_ lacI CRP+cAMP  P_lac2_ lacI CRP+cAMP  P_lac3_ lacI CRP+cAMP  P_lac4_ lacI CRP+cAMP  P_λ_ cI -----  P_tet1_ tetR -----  P_tet2_ tetR -----  P_ara_ araC -----  P_BAD_ araC + L-arabinose  P^1^(Constitutive) ---- -----  P^2^(Constitutive) ---- -----  **PROTEIN REPRESSOR**  lacI IPTG  tetR aTc |

**Table 2:** The activating and repressing interactions between the promoters and corresponding proteins and (/or) complexes.

**System of ordinary differential equations used in the first example:**

The ODE’s provide a mechanistic description that governs the time evolution of protein levels in the system. For example, in the first equation, ODE governing the production of *lacI* protein is provided. The binary variables Y_ij_, determine if a protein is expressed from a promoter. The mechanistic detail embedded in the first term of (1) is described below. A similar description has been adopted for deriving rest of the equations.

*lacI* protein suppresses the expression from *P_lac_* promoter in its tetrameric from. To this end, reactions (1) and (2) represent the dimerization and subsequent tetramarization of *lacI* along with the corresponding equilibrium constants. Reaction (3) represents the binding of *lacI* in its tetrameric from to *P_lac_* promoter. Reaction (4) represents the lumped description of the transcription and translation events that lead to expression of protein *P* from *P_lac_* promoter. Finally equation (5) represents the mass balance on the promoter regions in a cell.

Based on the above equations, the rate of production of protein *P* is given by

(6)

Assuming equations (1),(2) and (3) are fast and hence in equilibrium [1], we obtain the following equations.

Now combining (9) with (5), we obtain

(10)

With (10) and (6), we obtain

(11)

Assuming 1 promoter per cell and lumping the equilibrium constants *K1, K2, K3* together we get

(12)

| Parameter Description Value $\alpha_{\text{lac}}$ Transcriptional Efficiency 1.215  of P_lac_ promoter $\alpha_{\text{tet}}$ Transcriptional Efficiency 1.215  of P_tet_ promoter $\alpha_{\lambda}$ Transcriptional Efficiency 2.92  of P_λ_ promoter $\alpha_{\text{ara}}$ Transcriptional Efficiency 1.215  of P_ara_ promoter $K^{\lambda}$ Cumulative constant 0.33 nm^-2^  representing *cI* dimeration  and binding to P_λ_ promoter $K^{\text{tet}1}$ Cumulative constant 0.014 nm^-2^  representing *tetR* dimeration  and binding to P_tet_ promoter $K^{\text{tet}2}$ Cumulative constant 1.4 nm^-2^  representing *tetR* dimeration  and binding to P_tet_ promoter $K^{\text{lac}1}$ Cumulative constant 10 nm^-3^  representing *lacI* tetramerization  and binding to P_lac1_ promoter $K^{\text{lac}2}$ Cumulative constant 0.01 nm^-3^  and binding to P_lac2_ promoter |
| --- |

| Parameter Description Value $K^{\text{lac}2}$ Cumulative constant 0.01 nm^-3^ and binding to P_lac2_ promoter $K^{\text{lac}3}$ Cumulative constant 0.001 nm^-3^ representing *lacI* tetramerization  and binding to P_lac3_ promoter $K^{\text{lac}4}$ Cumulative constant 0.00001 nm^-3^ representing *lacI* tetramerization  and binding to P_lac4_ promoter $K^{\text{ara}}$ Cumulative constant 2.5 nm^-2^ representing *araC* dimerization  and binding to P_ara_ promoter $K^{\text{decay}}$ Decay rate of proteins 0.0693 s^-1^ – *cI,*  0.0346s^-1^ –*lacI, tetR*  0.0115s^-1^-*araC*  Decay rate of 0.0693 s^-1^  protein-inducer complex $K^{f}$ Association constant for  *lacI*-IPTG/*tetR-*aTc 0.05 nm^-1^s^-1^  binding $K^{b}$ Dissociation constant for  *lacI*-IPTG/*tetR*-aTc 0.1  binding |
| --- |

**Table 3:** Nominal Parameter Values used for the genetic toggle switch example

Rate of production terms employed for the Genetic Decoder and Concentration Band detector examples. See Equation 1.2.

Promoter (s): P_lac1_-P_lac4_

Promoter: P_λ_

Promoter : P_tet1_-P_tet2_

Promoter: P_ara_

Promoter: P_BAD_

Promoter: P^1^ (Constitutive Promoter)

Promoter: P^2^ (Constitutive Promoter)

Parameter Description Value Typical Range Reference/Comment

Min Max

$\alpha_{\text{lac}}$ Transcriptional Efficiency 2.15 10^-4^ 10 Assumed within range^a^

of P_lac_ promoters

$\alpha_{\text{tet}}$ Transcriptional Efficiency 2.15 10^-4^ 10 Assumed within range^a^

of P_tet_ promoter

$\alpha_{\lambda}$ Transcriptional Efficiency 2.15 10^-4^ 10 Assumed within range^a^

of P_λ_ promoter

$\alpha_{\text{ara}}$ Transcriptional Efficiency 1.215 10^-4^ 10 Assumed within range^a^

of P_ara_ promoter

$\alpha_{\text{BAD}}$ Transcriptional Efficiency 3.9 10^-4^ 10 Assumed within range^a^

of P_BAD_ promoter

$\alpha_{0}$ Basal Expression from 2.0 10^-4^ 10 Assumed within range^a^

P_lac_ promoters

$\alpha_{c}^{1}$ Constitutive Promoter 2.0 Assumed within range^a^

$\alpha_{c}^{2}$ Constitutive Promoter 2.15 Assumed within range^a^

[RNAP] Conc. Of RNA polymerase 30nm None Typical value

Parameter Description Value Typical Range Reference/Comment

Min Max

$K_{j}^{\text{decay}}$ Decay rate of proteins 0.0693 s^-1^ None t_1/2_ of ~10s

$K^{\lambda}$ Cumulative constant 0.33 nm^-2^ 10^-5^ 10 Estimated from [24]

representing *cI* dimeration

and binding to P_λ_ promoter

$K^{\text{tet}}$ Cumulative constant 0.14 nm^-2^ 10^-5^ 10 Estimated from [31]

representing *tetR* dimeration

and binding to P_tet_ promoter

$K^{\text{lac}(1)}$ Cumulative constant 10 nm^-3^ 10^-5^ 10 Estimated from [31]

representing *lacI* tetramerization

and binding to P_lac1_ promoter

$K^{\text{lac}(2)}$ Cumulative constant 0.01 nm^-3^ 10^-5^ 10 Estimated from [31]

representing *lacI* tetramerization

and binding to P_lac2_ promoter

$K^{\text{lac}(3)}$ Cumulative constant 0.001 nm^-3^  10^-5^ 10 Estimated from [31]

representing *lacI* tetramerization

and binding to P_lac3_ promoter

$K^{\text{lac}(4)}$ Cumulative constant 0.00001 nm^-3^ 10^-5^ 10 Estimated from [31]

representing *lacI* tetramerization

and binding to P_lac4_ promoter

$K^{\text{lac}1}$ Equlibrium constant 0.01 nm^-2^  10^-5^ 10 Typical Value

representing *CRP* dimerization

$K^{\text{lac}2}$ Equlibrium constant 0.01 nm^-2^ 10^-5^ 10 Typical Value

representing *CAMP* teramerization

$K^{\text{ara}1}$ Equlibrium constant 0.01 nm^-2^ 10^-5^ 10 Typical Value

representing *araC* dimerization

$K^{\text{ara}2}$ Equlibrium constant 0.01 nm^-2^ 10^-5^ 10 Typical Value

representing L-arabinose teramerization

$K^{\text{ara}}$ Cumulative constant 2.5 nm^-2^ 10^-5^ 10 Estimated from [31]

representing *araC* dimerization

and binding to P_ara_ promoter

^a^ Ranges provided in “The *Bacillus Subtilis Sin* Operon” An evolvable network motif; Voigt C A, D. M Wolf, A P Arkin (2005) Genetics March 169(3):1187-1202.

**Table 4:** Nominal parameter values used for the genetic decoder and concentration band detector studies.

- **Protein decay is assumed to be first order**
- **Constant Stimulus for Inducers at 40 nm**

Supplementary Figure 1: The sensitivity of the reporter proteins to varying levels of input signals. All concentrations are in nm. Absence of glucose implies presence of cAMP. Examination of the simulated levels reveals shown in the above figure reveals the following

- The GFP production is highly sensitive to levels of cAMP and L-arabinose. Even a slight increase in their levels results in elimination of GFP response.
- We find that while RFP expression is robust with respect to changes in cAMP level, it is highly sensitive to changes in L-arabinose.
- In contrast, BFP expression is robust to changes in L-arabinose and sensitive to changes in cAMP levels
- YFP expression is very sensitive to changes in both L-arabinose and cAMP levels.
- Overall these observations indicate that while this circuit is optimal with respect to the design variables or connectivity, the output protein levels exhibit sensitivity to input signals. This is an artifact of using “perfect promoters” without allowing for any leakiness further motivating the need to develop rational methods to safeguard against noise.

Supplementary Figure 2: Legend describing the representation adopted for the LOGIC gates.

Supplementary Information 3:

Consider an optimization problem (P) to minimize a function over a set.

(P)

where X represents the feasible region. The above problem (P) can be stated equivalently as following linear program (LP).

(LP)

Further, if *f*(.) is convex, then we have,

which represents the tangents or the supporting hyper planes of the objective function at all points in the feasible space.

In other words, problem (LP) attempts to enumerate the value of the objective function at all points within the feasible region to determine the minimum value. It clearly follows that to solve problem (LP) requires an exhaustive enumeration of all feasible points, which is a prohibitive exercise. To over come this problem, the outer approximation procedure, solves the problem (LP) iteratively by successively adding constraint at each iteration until local optimality is attained. The details of the constraint generation procedure can be found in [2-4]. Since, in the examples investigated in this work typically involve non-convex objective functions, we deployed this procedure multiple times to determine the best solution.

**References**

1.Hasty J, Isaacs F, Dolnik M, McMillen D, Collins J: Designer Gene Networks:

Towards Fundamental Cellular Control. Arxiv preprint physics/0103034 2001.

2. Duran, M.A. and I.E. Grossmann, "An Outer-Approximation Algorithm for a Class of Mixed-integer Nonlinear Programs," Math Programming 36, 307 (1986).

3. Grossmann, I.E., "Review of Nonlinear Mixed-Integer and Disjunctive Programming Techniques," Optimization and Engineering, 3, 227-252 (2002).

4. Floudas CA: *Nonlinear and Mixed-Integer Optimization: Fundamentals and Applications*: Oxford University Press; 1995.
